# Comparing pregnancy, childbirth, and neonatal outcomes in women with idiopathic polyhydramnios: a prospective cohort study

**DOI:** 10.1186/s12884-022-04625-y

**Published:** 2022-05-11

**Authors:** Raziyeh Vanda, Mahnaz Bazrafkan, Maryam Rouhani, Fatemeh Bazarganipour

**Affiliations:** 1 Department of Gynecology and Obstetrics, Yasuj University of Medical Sciences, Yasuj, Iran; 2 Social Determinants of Health Research Center, Yasuj University of Medical Sciences, Yasuj, Iran

**Keywords:** Perinatal, Idiopathic polyhydramnios

## Abstract

**Background:**

In this prospective cohort study, the perinatal outcome in idiopathic polyhydramnios compared with normal pregnancies was examined.

**Methods:**

This was a prospective cohort study of 180 singleton pregnancies who received care at the referral gynecology clinic of Yasuj, Iran between 2018–2020. The inclusion criteria comprised singleton pregnancies, gestational age > 34 weeks; fetuses without structural and chromosomal abnormalities in pregnancy screening test, no maternal diabetes, negative TORCH negative screening test, no Rh factor isoimmunization. Polyhydramnios was defined as: (i) amniotic fluid index ≥ 24 cm; (ii) maximal vertical pocket of ≥ 8 cm. Perinatal outcomes were recorded in both groups.

**Results:**

Postpartum hemorrhage (7.8% vs. 2.2; OR: 1.60; 95% CI 1.09–2.34) and cesarean delivery (51.11% vs. 21.11; OR: 1.88; 95% CI 1.42–2.50) and respiratory distress (4.4 v vs. 0; OR: 2.04; 95 C CI 1.75–2.80) was significantly higher in the idiopathic polyhydramnios (*P* ≤ 0.05) compared to normal pregnancy, which increased with severity of idiopathic polyhydramnios.

**Conclusion:**

In conclusion, the results of the current study, suggest that idiopathic polyhydramnios may be associated with a higher rate of postpartum hemorrhage, cesarean delivery, and respiratory distress than a normal pregnancy.

## Introduction

Amniotic fluid plays an important role in the normal growth of the fetus during pregnancy and childbirth and has certain consequences on the future health of children [1, 2]. The most common techniques used to measure ultrasound amniotic fluid volume include the maximum vertical pocket (MVP) or amniotic fluid index (AFI) [3]. The MVP is the vertical dimension (in centimeters) of the largest pocket of amniotic fluid that does not consistently contain the umbilical cord or fetal organs. AFI is calculated by dividing the uterus into four quadrants. The four maximum vertical diameters of the amniotic fluid pocket are summarized for the final index [3, 4]. Polyhydramnios has been identified as an abnormally large volume of amniotic fluid for gestational age and may be associated with a variety of fetal or maternal disorders. Risk factors of polyhydramnios include maternal diabetes, fetal heart failure, abnormal swallowing, and congenital infection [5].

In about 70% of cases, none of the above is considered a cause of polyhydramnios and is referred to as idiopathic condition [6–8]. However, the association between idiopathic polyhydramnios and the perinatal adverse outcome is not well recognized. In some studies, an association has been observed between idiopathic polyhydramnios and macrosomia infants for gestational age, the incidence of malpresentation, and cesarean delivery, but without an increase in other side effects [9–11]. In contrast, it has been shown in another studies, increase in perinatal mortality as well as a twofold increase in neonatal complications in the first year of life was reported [12, 13]. A relevant cohort study showed that pregnancies diagnosed with idiopathic polyhydramnios at the time of hospitalization and an increase in delivery at > 34 weeks, risk of induction of labor, cesarean delivery, shoulder dystocia, and operative vaginal delivery [14].

To date, idiopathic polyhydramnios is a matter of debate in obstetric practice, as perinatal outcomes idiopathic polyhydramnios is conflicting in literature and therefore this issue remains unsettled. It should be noted that no prospective study- related perinatal outcomes in idiopathic polyhydramnios were performed on this population. On the other hand, according to the above studies various complications have been reported, which gestational age is an important factor. Since prematurity is one of the most important causes of morbidity and mortality in infants, we restrict our population to gestational age ≥ 34 weeks (late prematurity) for controlling prematurity factors in our study outcomes.

Considering the above contradictory evidence, we aimed to perform the first prospective cohort studying the assessment of perinatal outcomes and idiopathic polyhydramnios in an Iranian population.

## Material and methods

### Design and data collection

This was a prospective cohort study of 180 singleton pregnancies who received care at the referral gynecology clinic of Yasuj, Iran between 2018–2020. It was approved by the ethics committee of Yasuj University of Medical Sciences Ethics Committee, Iran (IR.YUMS.REC.1398.150). Written informed consent was gained from all patients; all participants were informed that they could withdraw at any point during the study.

According to the study of Karahanoglu et al. [15] and α = 0.05, β = 0.2, P1 (C/S prevalence in case group) = 58, P2 (C/S prevalence in the control group) = 34.8, the sample size were estimated at least 83 women per group as followed.

$$n=\frac{{\left({Z}_{1-\alpha /2}+{Z}_{1-\beta }\right)}^{2}\left[{P}_{1}\left(1-{P}_{1}\right)+{P}_{2}\left(1-{P}_{2}\right)\right]}{{\left({P}_{1}-{P}_{2}\right)}^{2}}$$

The inclusion criteria were singleton pregnancies, gestational age > 34 weeks; fetuses without structural and chromosomal abnormalities in pregnancy screening test (anatomy scan) at 18–20 weeks of pregnancy, no maternal diabetes, negative TORCH negative screening test, no Rh factor isoimmunization to assure isolated polyhydramnios. Exclusion criteria include the unwillingness of the participants during sampling, having positive OGTT with 75 g in 26–30 weeks of pregnancy according to Iran Ministry of Health guideline.

### Description of study

The cohort was stratified into two groups: Exposure was defined as idiopathic polyhydramnios and defined as [16]: (i) amniotic fluid index (AFI) ≥ 24 cm; (ii) maximal vertical pocket (MVP) of ≥ 8 cm. Control that has normal AFI or MVP ( normal pregnancy). The severity of polyhydramnios stratified follows: mild ≥ 25–30 cm, moderate ≥ 30–35 cm, and severe ≥ 35 cm [17].

All of the participants underwent ultrasound examination by a single experienced clinician to confirm the diagnosis of polyhydramnios. If any congenital anomalies were detected antenatal, patients were excluded from the study. If a gestational diabetes mellitus diagnosis was confirmed, the patient was excluded from the study.

After presenting the purpose of the study to appropriate participants who met the inclusion criteria, written consent was obtained from each volunteer and divided into idiopathic polyhydramnios and control (AFI normal in ultrasound) groups. Perinatal outcomes were recorded in both the groups. All participants followed the prenatal care in our clinic under the supervision of one gynecologist who performed the project (RV). The pregnancy visit intervals were according to the Iran Ministry of Health guidelines.

### Measures

The following measures were evaluated in each group:

1. Demographic and reproductive information including age, gravid, occupation, and BMI were collected. BMI was estimated by dividing the patient’s weight by square of height (Kg/m^2^).
2. Obstetrical complications outcome including placenta abruption, preterm labor (34–37 weeks), postpartum hemorrhage, cesarean or NVD delivery, intrauterine fetal death (IUFD). Indications of CS were repeated cesarean delivery, cephalopelvic disproportion, fetal distress, malpresentation, macrosomia, failure to progress, placental abruption, HELLP syndrome, previous uterine surgery, placenta previa, patient request (elective choice), severe vulvar varicose vein, severe genital wart.
  We used California maternal quality care collaborative criteria for postpartum hemorrhage as followed [18]:
  Grade 1: Lost blood volume > 500ml after vaginal delivery and > 1000ml after cesarean delivery or change in vital signs more than 15% or heart rate > 110 beats per minute, blood pressure < 85/45mmHg.
  Grade 2: Persistent bleeding with lost volume > 1500ml
  Grade 3: Total blood volume > 1500ml or more than 2 units of pack cell transfusion, unstable vital signs, or suspected diffuse intravascular coagulation.
3. Neonatal outcomes including anthropometric characteristics (weight, height, and head circumference), low birth weight (< 2500 g), macrosomia (> 4000 g), fifth-minute Apgar score, neonatal death within the first 7 days, NICU admission, respiratory disease syndrome (RDS). Physical findings of RDS in the affected infant include moaning, tachypnea (breathing more than 60 beats per minute), intercostal and subcostal retraction, cyanosis, and nasal flap jumps. Radiological findings include bilateral diffuse reticulogranular images or a ground glass view, the presence of an air bronchogram, a normal heart size, or a small size due to insufficient dilation of the lungs. Laboratory findings include respiratory acidosis due to alveolar atelectasis. Metabolic acidosis is also caused by lactic acidosis due to inadequate tissue perfusion and hypoxia due to right-to-left shunt. The diagnosis is made by a neonatologist.

In mild cases of idiopathic polyhydramnios, simple control and follow-ups, continuous ultrasound, and conservative treatment methods are recommended [19]. The present study did not require any treatment process for this group. In cases with moderate to severe idiopathic polyhydramnios and maternal distress and gestational age less than 37 weeks, hospitalization and partial treatment measures to alleviate the mother's discomfort like amnioreduction or pharmacological intervention, the patient’s consent and physician’s assessment of the scenario varied. In this study, we conducted the intervention using fetal symptoms (including enlarged uterus pressing on the cervix which causes preterm delivery) in addition to maternal symptoms based on the measurement of cervical length and cervix funneling. We followed the approach of previous protocols in which the administration of indomethacin at a dose of 1.5 mg per body weight per day for 1 week is prescribed for pregnancies less than 32 weeks [20]. In terms of gestational age (38–42 weeks), induction of labor / cesarean section was performed (depending on the indications of the obstetrician e.g. fetal distress). Considering our inclusion criteria(i.e. gestational age > 34 weeks of pregnancy), referring to patient's history, up to 200 mg of indomethacin was prescribed for 14 patients (100%) in case the group with moderate- severe polyhydramnios at a gestational age of less than 32 weeks. Amnioreduction was conducted in 2 patients (14.28%) in the case group with moderate- severe polyhydramnios. All patients with mild polyhydramnios were managed without treatment (expected management) until the term. All parameters were compared within groups (Tables 2 and 3).

### Statistical analysis

Demographic data of the groups were expressed as mean ± SD or number (percentage) and a comparison of these data was performed by × 2, Fisher, U-Manwithney, and t-test. The normality of the distributions was tested using the Kolmogrov-Smirnov test. Logistic regression was specified to evaluate obstetric and neonatal outcomes in patients between two groups. Confounding factors were gestational age, BMI, age, gravid. Results from the final model are presented as an odds ratio with a 95% confidence interval. *P* values were set as 0.05 for all analyses. The statistical program for Social Sciences (SPSS, version 21; SPSS, Chicago, IL). There were no missing values. Therefore, no missing imputation technique was used. This manuscript was prepared under STROBE guidelines for observational studies.

## Results

### Baseline characterizes of participant

During the study period, a total of 180 patients were included. Patient characteristics were presented in Table 1. There were no statistically significant differences between the two groups according to age, BMI, occupation, and gravid.

**Table 1:** Socio-demographic and clinical characteristic between groups

Variable	Idiopathic polyhydramnios *N* = 90	Normal pregnancy *N* = 90	*P* value
Age^a^	28.32 ± 5.79	27.5 ± 5.57	0.57
Occupation^b^			
Occupied	5(5.6)	3(3.33)	0.10
Housewife	85(94.44)	87(96.66)	
BMI^a^	26.4 ± 3.65	24.4 ± 6.76	0.52
Gravid^a^	1.24 ± 0.31	1.27 ± 0.69	0.37

^a^Mean ± SD, ^b^N(%)

### Obstetric outcomes between groups

Table 2 compares obstetric outcomes between the two groups. Results show that there is no significant differences between the two groups in preterm birth in 34–37 weeks of pregnancy (*P* > 0.05).

**Table 2:** Obstetrics outcomes between groups

Variable	Normal pregnancy *N* = 90	Idiopathic polyhydramnios *N* = 90	Severity of Idiopathic polyhydramnios		*P* value
			Mild (*n* = 76)	Moderate to severe(*n* = 14)	
Preterm labor in 34–37 weeks of pregnancy^a^	0(0)	1(1)	0	0	0.08
Gestational age^b^	277.94 ± 7.60	274.87 ± 8.93	275.18 ± 9.01	273.0 ± 8.21	0.08
Cesarean delivery ^a^	19 (21.11))	46(51.11)	34(47.73)	12(85.71)	< 0.001^c^
Postpartum hemorrhage^a^	2(2.2)	7(7.8)	5(6.58)	2(14.29)	0.05^c^
Postpartum hemorrhage severity^a^					
Grade 1	0	1(1.1)	1(1.31)	0	0.05^c^
Grade 2	0	5(5.5)	3(3.94)	2(14.29)	
Grade 3	2(2.2)	1(1.1)	1(1.31)	0	

^a^N(%), ^b^Mean(SD)

^c^Significant diffrence between subgroup of idiopathic polyhydramnios (mild vs. moderate to severe severity)

Moreover, the gestational age at termination of pregnancy did not differ between the two groups (277.94 ± 7.60 vs. 274.87 ± 8.93, *p* = 0.08).

The postpartum hemorrhage and cesarean delivery were significantly higher in the idiopathic polyhydramnios compared to normal pregnancy (*P* ≤ 0.05), which increased with the severity of idiopathic polyhydramnios.

Indication for caesarean section is shown in Table 3. Most cesarean births in the case group resulted from a prior cesarean history and failure to progress; however, in the control group, most of the caesarean births resulted from prior cesarean birth and then CPD.In a subgroup of idiopathic polyhydramnios, the most cesarean births resulted from prior caesarean history and failure to progress;

**Table 3:** Indication of cesarean between groups

Variable^a^	Idiopathic polyhydramnios *N* = 46	Normal pregnancy *N* = 19	Severity of Idiopathic polyhydramnios	
			Mild (*n* = 30)	Moderate to severe(*n* = 16)
Previous history of cesarean	21(45.65)	7(36.84)	18(60)	3(18.75)
Fetal distress in labor	10(21.73)	7(36.84)	6(20)	4(25)
Mal-presentation	1(2.18)	1(5.26)	1(3.33)	0
Failure to progress	11(23.91)	1(5.26)	6(20)	5(31.25)
CPD	2(4.34)	2(10.52)	2(6.66	0
Others^b^	1(2.18)	1(5.26)	1(3.33)	

^a^N(%)

^b^placental abruption, HELLP syndrome, previous uterine surgery, placenta previa, patient request(elective choice), severe vulvar varicose vein, severe genital wart

We did not have any IUFD and placenta abruption cases in two groups. It should be noted that we did not identify any obvious fetal abnormalities subsequently until birth in two groups.

### Neonatal outcomes between groups

Neonatal characteristics were presented in Table 4. The findings of this study showed that there were no significant differences in neonate weight, height and head circumstance, low birth weight, neonatal death, macrosomia, and neonatal admission in NICU (*P* > 0.05). All the neonate in the two groups had neonatal Apgar score 7–10 (*P* > 0.05).

**Table 4:** Neonatal outcome between groups

Variable	Normal pregnancy *N* = 90	Idiopathic polyhydramnios *N* = 90	Severity of Idiopathic polyhydramnios		*P* value ^c^
			Mild (*n* = 76)	Moderate to severe(*n* = 14)	
Macrosomia ^a^	4(4.44)	11(12.22)	10(13.15)	1(7.14)	0.07
Low birth weight ^a^	4(4.44)	1(1.11)	1(1.31)	0(0)	0.21
Neonate weight ^b^	3298.43 ± 86.08	3461.95 ± 0.44	3482.76 ± 0.85	3342.85 ± 0.37	0.02
Neonate height ^b^	51.07 ± 6.31	50.97 ± 3.11	51.10 ± 2.73	50.28 ± 4.77	0.32
Neonate head circumstance ^b^	35.33 ± 3.11	35.2 ± 2.34	35.48 ± 2.47	35.70 ± 1.55	0.64
Respiratory distress ^a^	0(0)	4(4.44)	1(1.31)	3(21.42)	0.04^c^
NICU admission ^a^	1(1.11)	2(2.22)	1(1.31)	1(7.14)	0.59
Neonatal dead ^a^	1(1.11)	1( 1.11)	1(1.31)	0	-

^a^N(%), ^b^Mean( SD)

^c^Significant diffrence between subgroup of idiopathic polyhydramnios (mild vs. moderate to severe severity)

The respiratory distress was significantly higher in the idiopathic polyhydramnios compared to normal pregnancy (*P* ≤ 0.05). (Table 4), which increased with the severity of idiopathic polyhydramnios.

### Regression model

In the present study, we found that women with idiopathic polyhydramnios had a more increased odds of postpartum hemorrhage (7.8% vs. 2.2; OR: 1.60; 95% CI 1.09–2.34), a cesarean delivery (51.11% vs. 21.11; OR: 1.88; 95% CI 1.42–2.50) and respiratory distress (4.4% vs. 0%; OR: 2.04; 95% CI 1.75–2.80) compared to normal pregnancy in the logistic regression model (Table 5).

**Table 5:** The result of logistic regression test

	*p* value	SE	odds ratio	95% CI	
				Lower	Upper
Cesarean delivery	< 0.001	1.25	2.8	1.42	2.50
Postpartum hemorrhage	0.05	1.02	2.4	1.09	2.34
Respiratory distress	0.04	0.39	2.04	1.75	2.80

Hosmer and Lemeshow test *p* = 0.89

## Discussion

Polyhydramnios is present in about 2% of pregnancies and has been associated with adverse pregnancy outcomes. Several retrospective studies have examined the effect of idiopathic polyhydramnios on neonatal outcomes, but so far to our knowledge, no prospective cohort study has been performed. The current analysis is mainly highlighting two major perinatal outcomes that may accompany idiopathic polyhydramnios cases; as they are high postpartum hemorrhage and cesarean delivery. In idiopathic polyhydramnios, postpartum hemorrhage was 1.6 times more prevalent compared with the normal pregnancy group, which was 7.8% as compared to 2.2% for women with normal AFI. Excess postpartum hemorrhage observed with idiopathic polyhydramnios is biologically acceptable because increased fluid levels and fetal size may cause exaggerated uterine traction and increased uterine atony. Unlike the studies reported on different types of polyhydramnios [21]. This finding is consistent with previous studies on increased postpartum hemorrhage in pregnancies with idiopathic polyhydramnios [22, 23].

In this study, we found that idiopathic polyhydramnios had effects on delivery mode. In idiopathic polyhydramnios, C/S was 1.88 times more common than in normal pregnancy, which was 51.11% as compared to 21.11% for women with normal AFI. This influence seems to progress with polyhydramnios severity. Most caesarean births in the case group resulted from a prior caesarean history and failure to progress; however, in the control group, most of the caesarean births resulted from prior caesarean birth and then CPD. This finding is consistent with previous studies about increased rates of primary C/S in pregnancies with idiopathic polyhydramnios [24–27], however, other studies did not report this association [28, 29], overall incidence in the literature varies between 22 and 35% [30]. Obviously, study population is too small to be conclusive.

In this study, respiratory distress rate was significantly higher in the idiopathic polyhydramnios compared to normal pregnancy. The rate of fetal distress in the experimental group was 4.4% compared to the control group at 0%. Respiratory distress was 2.04 times more common in idiopathic polyhydramnios than that of the controls in four previous studies [26, 31–33].

To our best knowledge, this is the first study attempted to compare the perinatal outcomes in idiopathic polyhydramnios with normal a pregnancy in a prospective cohort study. The results of the study led us that idiopathic polyhydramnios should be considered as an important finding, which can result in maternal and perinatal complications. Thus, despite some other studies [24, 34], we believe that these mothers need more prenatal care and fetal monitoring. Our study has several strengths including prospective nature and measurement of AFI by one clinician which decrease the bias. Moreover, it seems that the sample size in this study was quite large comparing to previous studies and the study power of 0.9 is adequate. Finally, we used standard measurements for the accurate assessment outcomes and the inclusion of the control group for comparison.

There are some limitations. First, infant data after 1 year are also not available. Dorleijn et al. Reported that 28.4% of infants were diagnosed with idiopathic polyhydramnios during the first year of life, which emphasizes the importance of following up these infants [26]. Morover, although we exclude gestational diabetes according to positive OGTT with 75 g in 26–30 weeks of pregnancy according to the Iran Ministry of Health guidelines, in some of the cases of “idiopathic polyhydramnios” it may not be idiopathic, but could be underlying gestational diabetes. But, the managment in the two groups of study about screening were similar and based on national guildines. We used unique observer for our sonography studies.

Therefore, inter—and intra observer variability was not formally assessed in this study among the one our sonographer performing measurements for the women participants in the study population. However, any errors are likely to have been randomly distributed between the exposed and naive cases. In this study the gestational age for the diagnosis of the idiopathic polyhydramnios is not controlled. However, all women in the two groups were carefully evaluated for inclusion in the study at 34 weeks of pregnancy where they were included regardless the age of diagnosis of polyhydramnios in the study if they were eligible. It is recommended that this issue be considered in future studies. Moreover, we restrict our population to gestational age ≥ 34 weeks (late prematurity) for controlling prematurity factor in our study outcomes. Finally, the study was limited as it was a single center study.

## Conclusion

Accordingly, in the current study, idiopathic polyhydramnios was associated with maternal and fetal complications and increased perinatal complications, thus undermining the fact that pregnancies due to idiopathic polyhydramnios are at higher risk and should therefore be closely monitored. According to this study, idiopathic idiopathic polyhydramnios may be increases the risk of postpartum hemorrhage, cesarean delivery, and respiratory distress. So, it is better patients with polyhydramnios closely monitored and followed up.

## Data Availability

The primary data for this study is available from the authors (RV) on direct request.

## Abbreviations

AFI: Amniotic fluid index
MVP: Maximal vertical pocket
PIH: Pregnancy induced hypertension
SGA: Small for gestational age

## References

[1] Blencowe H, Cousens S, Jassir FB (2016). National, regional, and worldwide estimates of stillbirth rates in 2015, with trends from 2000: a systematic analysis. Lancet Glob Health.

[2] Low JA, Pickersgill H, Killen H (2001). The prediction and prevention of intrapartum fetal asphyxia in term pregnancies. Am J Obstet Gynecol.

[3] Coombe-Patterson J. Amniotic Fluid Assessment: Amniotic Fluid Index Versus Maximum Vertical Pocket. J Diagnostic Medical Sonography. 2017;33(4):280–3. 10.1177/8756479316687269.

[4] Phelan JP, Ahn MO, Smith CV, Rutherford SE, Anderson E. Amniotic fluid index measurements during pregnancy. J Reprod Med Obstet Gynaecol. 1987;32(8):601–4. 3309290

[5] Amniotic Fluid Problems/Hydramnios/Oligohydramnios. Children’s Hospital of Philadelphia. https://www.chop.edu/conditions-diseases/amniotic-fluid-problemshydramniosoligohydramnios. Accessed 26 Mar 2020.

[6] Panting-Kemp A, Nguyen T, Chang E, Quillen E, Castro L (1999). Id-iopathic polyhydramnios and perinatal outcome. Am J Obstet Gynecol.

[7] Desmedt EJ, Henry OA, Beischer NA (1990). Polyhydramnios and asso-ciated maternal and fetal complications in singleton pregnancies. Br J Obstet Gynaecol.

[8] Many A, Hill LM, Lazebnik N, Martin JG (1995). The association be-tween polyhydramnios and preterm delivery. Obstet Gynecol.

[9] Smith CV, Plambeck RD, Rayburn WF, Albaugh KJ (1992). Relation of mild idiopathic polyhydramnios to perinatal outcome. Obstet Gynecol.

[10] Panting-Kemp A, Nguyen T, Chang E, Quillen E, Castro L (1999). Idiopathic polyhydramnios and perinatal outcome. Am J Obstet Gynecol.

[11] Chauhan SP, Martin RW, Morrison JC (1993). Intrapartum hydramnios at term and perinatal outcome. J Perintol.

[12] Pri-Paz S, Khalek N, Fuchs KM, Simpson LL (2012). Maximal amniotic fluid index as a prognostic factor in pregnancies complicated by polyhydramnios. Ultrasound Obstet Gynecol.

[13] Maymon E, Ghezzi F, Shoham-Vardi I (1998). Isolated hydramnios at term gestation and the occurrence of peripartum complications. Eur J Obstet Gynecol Reprod Biol.

[14] Aviram A, Salzer L, Hiersch L (2015). Association of isolated polyhydramnios at or beyond 34 weeks of gestation and pregnancy outcome. Obstet Gynecol.

[15] Karahanoglu E, Ozdemirci S, Esinler D, Fadıloglu E, Asiltürk S, Kasapoglu T, et al.Intrapartum, postpartum characteristics and early neonatal outcomes of idiopathic polyhydramnios. J Obstet Gynaecol. 2016;36(6):710–4. 10.3109/01443615.2016.1148126 26926000

[16] Lazebnik N, Many A (1999). The severity of polyhydramnios, estimated fetal weight and preterm delivery are independent risk factors for the presence of congenital malformations. Gynecol Obstet Invest.

[17] Rock gohn A, Jones Howard W. Telinds operative gynecology. Ninth edition . lippincott williams & willkins. 2003: chap: 21, 485–498.

[18] Crowther CA, Haslam RR, Hiller JE, Doyle LW, Robinson JS, Australasian Collaborative Trial of Repeat Doses of Steroids (ACTORDS) Study Group (2006). Neonatal respiratory distress syndrome after repeat exposure to antenatal corticosteroids: a randomised controlled trial. Lancet.

[19] Cabrol D, Jannet D, Pannier E (1996). Treatment of symptomatic polyhydramnios with indomethacin. Eur J Obstet Gynecol Reprod Biol.

[20] Golan A, Wolman I, Sagi J, Yovel I, David MP (1994). Persistence of polyhydramnios during pregnancy — its significance and correlation with maternal and fetal complications. Gynecol Obstet Invest.

[21] Lallar M, Anam Ul H, Nandal R (2015). Perinatal Outcome in Idiopathic Polyhydramnios. J Obstet Gynaecol India.

[22] Wiegand SL, Beamon CJ, Chescheir NC, Stamilio D (2016). Idiopathic Polyhydramnios: Severity and Perinatal Morbidity. Am J Perinatol.

[23] Panting-Kemp A (1999). Idiopathic polyhydramnios and perinatal outcome. Am J ObstetGynecol.

[24] Maymon E (1998). Isolated hydramnios at term gestation and the occurrence of peripartum complications. Eur J Obstet Gynecol Reprod Biol.

[25] Chen KC (2005). Perinatal outcomes of polyhydramnios without associated congenital fetal anomalies after the gestational age of 20 weeks. Chang Gung Med J.

[26] Dorleijn DM (2009). Idiopathic polyhydramnios and postnatal findings. J Matern Fetal Neonatal Med.

[27] Taskin S (2013). Perinatal outcomes of idiopathic polyhydramnios. Interv Med ApplSci.

[28] Giannella L, Mfuta K, Pedroni D (2013). Delays in the delivery room of a primary maternity unit: a retrospective analysis of obstetric outcomes. J Matern Fetal Neonatal Med.

[29] Dorleijn D, Cohen-Overbeek T, Groenendaal F, Bruinse H, Stoutenbeek P (2009). Idiopathic polyhydramnios and postnatal find-ings. J Matern Fetal Neonatal Med.

[30] Khan S, Donnelly J (2017). Outcome of pregnancy in women diagnosed with idiopathic polyhydramnios. Aust N Z J Obstet Gynaecol.

[31] Chen KC, Liou JD, Hung TH (2005). Perinatal outcomes of pol-yhydramnios without associated congenital fetal anomalies after the gestational age of 20 weeks. Chang Gung Med J.

[32] Maymon E (1998). Isolated hydramnios at term gestation and the occurrence of peripartum complications. Eur J Obstet Gynecol Reprod Biol.

[33] Smith CV (1992). Relation of mild idiopathic polyhydramnios to perinatal outcome. ObstetGynecol.

[34] Clark S, Blum J, Blanchard K (2002). Misoprostal use in Obsterics and gynecology in Brazil, Gamaica, the united states. Int J Gynaecol Obstet.

